# Monte Carlo Modeling of Shortwave-Infrared Fluorescence Photon Migration in Voxelized Media for the Detection of Breast Cancer

**DOI:** 10.3390/diagnostics10110961

**Published:** 2020-11-17

**Authors:** Tatsuto Iida, Shunsuke Kiya, Kosuke Kubota, Takashi Jin, Akitoshi Seiyama, Yasutomo Nomura

**Affiliations:** 1 Department of Systems Life Engineering, Maebashi Institute of Technology, Maebashi 371-0816, Japan; m1956001@maebashi-it.ac.jp (T.I.); m1771013@maebashi-it.ac.jp (S.K.); m1771014@maebashi-it.ac.jp (K.K.); 2 Laboratory for Nano-Bio Probes, RIKEN Center for Biosystems Dynamics Research, Suita 565-0874, Japan; tjin@riken.jp; 3 Human Health Sciences, Graduate School of Medicine, Kyoto University, Kyoto 606-8507, Japan; seiyama.akitoshi.7x@kyoto-u.ac.jp

**Keywords:** shortwave-infrared light, near-infrared light, visible light, fluorescence, breast cancer, duct, visible human project, Monte Carlo simulation, voxelized media

## Abstract

Recent progress regarding shortwave-infrared (SWIR) molecular imaging technology has inspired another modality of noninvasive diagnosis for early breast cancer detection in which previous mammography or sonography would be compensated. Although a SWIR fluorescence image of a small breast cancer of several millimeters was obtained from experiments with small animals, detailed numerical analyses before clinical application were required, since various parameters such as size as well as body hair differed between humans and small experimental animals. In this study, the feasibility of SWIR was compared against visible (VIS) and near-infrared (NIR) region, using the Monte Carlo simulation in voxelized media. In this model, due to the implementation of the excitation gradient, fluorescence is based on rational mechanisms, whereas fluorescence within breast cancer is spatially proportional to excitation intensity. The fluence map of SWIR simulation with excitation gradient indicated signals near the upper surface of the cancer, and stronger than those of the NIR. Furthermore, there was a dependency on the fluence signal distribution on the contour of the breast tissue, as well as the internal structure, due to the implementation of digital anatomical data for the Visible Human Project. The fluorescence signal was observed to become weaker in all regions including the VIS, the NIR, and the SWIR region, when fluorescence-labeled cancer either became smaller or was embedded in a deeper area. However, fluorescence in SWIR alone from a cancer of 4 mm diameter was judged to be detectable at a depth of 1.4 cm.

## 1. Introduction

Ductal carcinoma is the most common type of breast cancer and tends to progress to invasive cancer. Breast conservation therapy should be considered when the cancer is less than 2.0 cm [1,2]. However, it was shown by the accuracy of the same MRI(magnetic resonance imaging)-sonography co-registration system that the mean lesion size correlated well on MRI (11.4 mm; range 6–28 mm) compared with that of sonography (10.3 mm; range 6–28 mm) [3]. Therefore, early breast cancers of only several millimeters have occasionally failed to be detected by the use of such noninvasive methods alone. However, shortwave-infrared (SWIR) fluorescence imaging for the detection of small cancers offers a higher contrast and sensitivity with deeper penetration depths in comparison with the conventional visible (VIS) and the near-infrared (NIR) fluorescence imaging, thus it has attracted much attention recently [4,5]. As radiation exposure due to SWIR is much less than that from X-rays in mammography and gamma-rays in PET, SWIR fluorescence imaging can be used repetitively, which suggests its suitability in the case of young subjects. Furthermore, it would have a higher spatial resolution than the sentinel lymph-node biopsy with VIS (Patent blue) or NIR fluorescence (Indocyanine green, ICG) because of the lower scattering property [6,7,8,9]. Indeed, a fluorescent image of a small breast cancer of several millimeters was obtained from small animal experiments [10].

Before clinical application, detailed numerical analyses of the behavior of excitation and emission photons are required, since optical parameters such as scattering and absorption coefficients differ between humans and small experimental animals. In our previous study, as the first step for the detection of early breast cancer, Monte Carlo modeling in a multi-layered media (MCML) for fluorescence photon migration of the ICG in the NIR was proposed [11]. In contrast to the VIS fluorescence of fluorescein, the NIR fluorescence of the ICG showed effectiveness in detecting a cancer of 1.0 cm in diameter at a depth of 1.0 cm. This suggests that smaller cancers are probably detected when SWIR fluorescence is utilized. However, the analytical model was composed of a spherical cancer with fluorescence in the fat layer below the flat skin surface. The results of the analysis in the approximate model of the layered structure are not always correct, because actual breast tissue has a complex three-dimensional structure, e.g., the mammary gland. Therefore, taking the high spatial resolution of the SWIR fluorescence imaging into account, an analytical model which reflected the contour of the breast surface and its internal structure was pursued. Recently, Monte Carlo modeling in voxelized media (MCVM), by which the authors could track photon migration in realistic brain tissue structure, was reported by Li et al. [12,13], where a digital anatomical dataset of the voxelized media of the Monte Carlo model was implemented in which optical parameters were set specific to regions of the human tissue. Here we developed their method to apply to a breast cancer model, as follows.

The authors predicted distribution within the tissue of light absorption in the VIS and the NIR. In this study, we developed MCVM for the analysis of excitation and emission. The intensity of fluorescence is proportional to the concentration of the fluorescent molecules as well as the excitation efficiency. The accumulation of fluorescent probes in the cancer is dependent on the expression level of the marker protein. The concentration of fluorescent molecules located in the cancer was assumed to be spatially constant. On the other hand, it was quite difficult to set the excitation efficiency to be spatially constant. When incident photons reached the cancerous cell, the fluorescent molecules within it partially absorbed the energy. Therefore, the excitation efficiency was influenced by the optical properties surrounding the cancer and its depth. When optical parameters were set, specific to the regions of the duct with a complex morphology and the fat tissue, photon migration in association with excitation and emission would reflect the internal structure faithfully. Thus, the feasibility of detecting early breast cancer was examined by analyzing the SWIR fluorescence, which was emitted by several mechanisms, using MCVM with exact distribution of the optical parameters.

## 2. Materials and Methods

### 2.1. Breast Model

**Figure 1:**
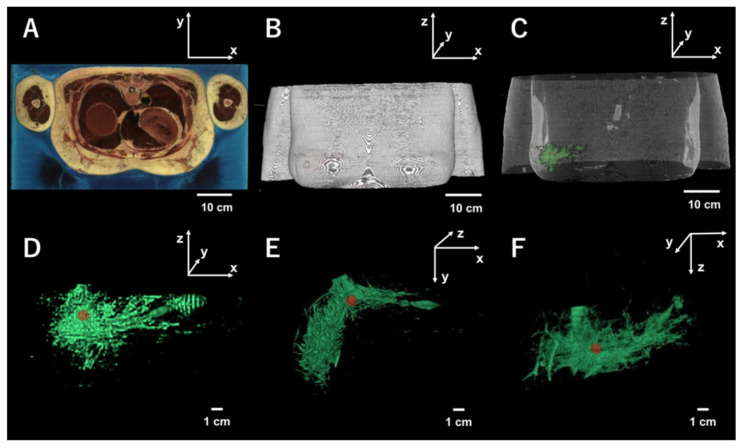
Visualization of the internal structure of the female breasts from the Visible Human Project (VHP). (**A**) Slice of the upper body. (**B**) Three-dimensional reconstruction of the upper body. (**C**) Internal structure in the reconstruction. (**D**) Enlarged internal structure with fluorescence-labeled cancer with 1 cm diameter. (**E**) The 3-D vision of the enlarged structure is observed from a different angle. (**F**) 3-D vision from another different angle. See text.

Digital anatomical data in which the internal structure of the breast was reflected were pursued for the improvement of our previous Monte Carlo model [11,14,15,16]. In the previous studies, Li et al. used a dataset of cross-sectional cryosection images which was provided by the Visible Chinese Human (VCH) [12,13]. However, due to limited access, there was a difficulty in obtaining the dataset from VCH. Therefore we used a public-domain library of cross-sectional cryosection, CT, as well as MRI images provided by the Visible Human Project (VHP) of the US National Library of Medicine [17]. Because 3D tomography of the breast was obtained from the dataset, it permitted us to analyze photon behaviors in the Monte Carlo model set to a complex internal structure identical to the human breast. In this study, the thoracic part of an anatomical dataset built from a cadaver of an American woman who had died of heart disease at the age of 59 was used. With the permission of her husband, the body was frozen, thinly sliced more than 5000 times, and photographed. The thickness of each slice was 0.33 mm. The three-dimensional reconstruction of the thoracic part, built from thin slices as in Figure 1A, is shown in (B). When images of the slice were processed based on the anatomy of the breast as described in the next section, the mammary gland (green structure with complex morphology) was included within the thoracic part (C). As shown in (D), fluorescence-labeled cancer (red sphere) was set in the duct of the structure. To indicate the structure observed from a different angle, rotating (D) π/2 clockwise on the *x*-axis was performed to obtain (E). Furthermore, (F) was obtained by rotating (D) π/2 once more. 

### 2.2. Image Processing and Implementation of the Model

**Figure 2:**
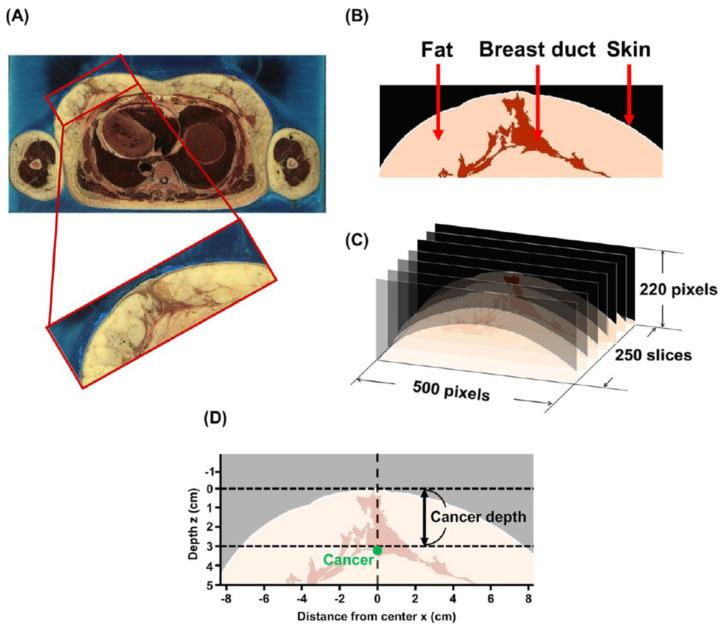
Implementation of the contour and the internal structures derived from breast anatomical data of the Visible Human Project (VHP). (**A**) Cropped image from a slice. (**B**) Allocation of the fat, the duct and the skin based on pixel value. (**C**) Reconstruction of a 3-D voxel model. (**D**) Setting of coordinate system in the slice containing the center and top of nipple (*x* = 0, *z* = 0). The depth denotes the distance from *z* = 0 to the upper surface of the cancer.

**Figure 3:**
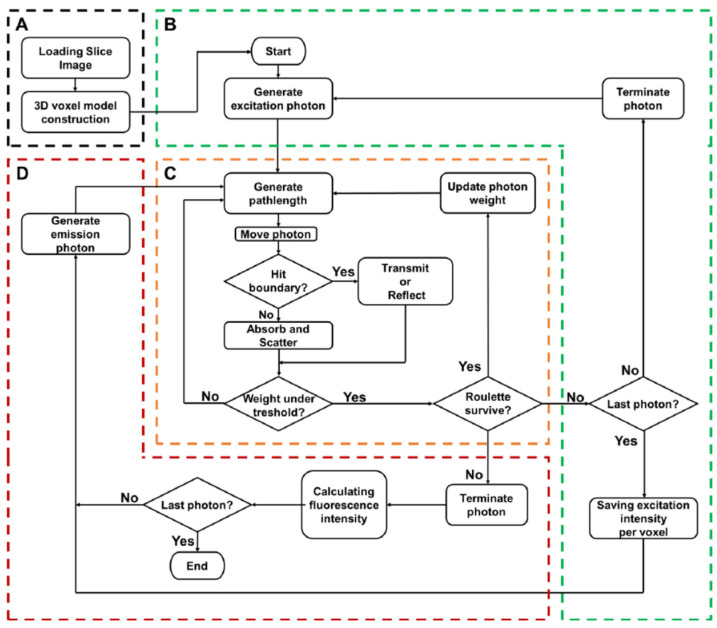
Flowchart of the Monte Carlo simulation in voxelized media for breast tissue. (**A**) Implementation of the breast anatomical data of the VHP. (**B**) Excitation part of the simulation. (**C**) Computation routines were based on Wang et al. [25]. (**D**) Emission part.

250 slices were used from the thoracic part, with 2048 × 1216 pixels for each slice provided by the VHP, where the breast area of 500 × 220 pixels was cropped using an image processing software (ImageJ). A cropped breast image (24-bit RGB color) from the original VHP image is shown in Figure 2A. Regarding the pixel values, the cropped image in Figure 2B was allocated to areas of fat of more than 180, as well as to areas of the breast duct between 80 and 120, respectively, after conversion to an 8-bit grayscale image. The boundary between the outside and inside tissue area was extracted and the border of two pixels width was assumed to be the skin layer. Previous studies were used to set the optical parameters specific to the three areas in Table 1 [18,19,20,21,22]. In this study, taking medical applications into account, the excitation and emission wavelengths of the fluorescein (excitation max (Ex) 488 nm, emission max (Em) 520 nm, quantum yield (QY): 0.95, concentration (C): 0.75 mM, absorption coefficient (εC): 12 cm^−1^) were selected, which were used for the examination of the fundus in the VIS region, as well as those of the ICG (Ex 780 nm/Em 820 nm, QY: 0.09, C: 0.2 mM, εC: 20 cm^−1^) for the sentinel lymph-node biopsy in the NIR region [6,7,8,9,23,24]. Fluorescence imaging in the VIS region is an effective diagnostic method for transparent media such as the eyeball, but it is difficult to use in turbid media which is characteristic of the optical properties of breast tissue. For SWIR imaging, we employed lead sulfide (PbS) quantum dots (QDs) (Ex 970 nm/Em 1100 nm, QY: 0.40, C: 1.0 µM, εC: 0.63 cm^−1^) as fluorescent probes which have been used in prior animal experiments despite the lack of medical applications of QDs [5]. As shown in Figure 2C and Figure 3A, the optical parameters were implemented in the voxel of the 3-dimensional matrix (500, 250, 220). Each voxel was of 0.033 × 0.033 × 0.033 cm^3^. The fluorescence-labeled cancers with a diameter of 1, 4, 7, and 10 mm were embedded at a depth of 1 to 3 cm (Figure 2D).

### 2.3. The Monte Carlo Simulation

The software for Monte Carlo modeling of fluorescence was developed with the use of the C programing language within the Microsoft Visual Studio program. As shown in Figure 3B, this simulation was executed by the input of an excited photon in the voxelized media, where each voxel had optical parameters specific to the three areas of the skin, duct, and fat. In Figure 3C, the computation routines described by Wang et al. were used [25]. In brief, each pathlength was considered to be the distance traveled by a photon from the original position to the next interaction site between the photon and the tissue, due to scattering and absorption. The pathlength and the direction were calculated using a random number, anisotropy *g*, as well as the total attenuation coefficient *μ_t_*, which is the sum of the scattering coefficient *μ_s_* and the absorption coefficient *μ_a_*. Although each excitation photon had an initial weight of unity, the original weight of the photon was reduced by *μ_a_*/*μ_t_* during movement of the photon to the next site. Pseudo-random numbers generated using the Mersenne twister method were used [26]. Photons were reflected or transmitted based on Fresnel reflectance, calculated using refractive indices n of each layer when they crossed the boundary between two layers [25]. Random paths for photons were computed until the weight of each photon was absorbed to less than a threshold, or the photon left the medium.

The photon weight absorbed within each voxel was scored after completion in order to calculate the behavior of a million excitation photons. This was divided by *μ_a_* of the voxel to obtain the fluence. The fluence can be considered as the weight of photons passed and the amount of energy passed per unit area (W/cm^2^). Furthermore, since the fluence yielded a response to a light source 1 W/cm^2^, it was converted into the response to 50 mW/cm^2^ of light source, generally used in tissue spectroscopy [13,27]. The fluence was inherited by the emission photon in two ways as shown in Figure 3D. In the previous study, without the excitation gradient [14], the weight of the emission photon was set as spatially constant by the use of excitation fluency in the center of the cancer. In the present study with an excitation gradient, the emission photon had an initial weight dependent on excitation fluency in each voxel. A million emission photons were generated isotopically in a random position within the cancer.

## 3. Results and Discussion

### 3.1. Excitation Gradient

In the previous study [11], we used the fluorescence Monte Carlo model in which spherical cancer was embedded in the fat layer below the flat skin surface for detecting of early breast cancer. Fluorescence was based on two assumptions. (1) The fluorescent probes were distributed within cancer homogeneously. (2) All probes were excited by the same intensity as the light which reached the center of the cancer. Compared with cancer near the body surface, weaker excitation light reached cancer embedded deeply. There may have been a difference in excitation light intensity between cancer in shallow and deep regions.

**Figure 4:**
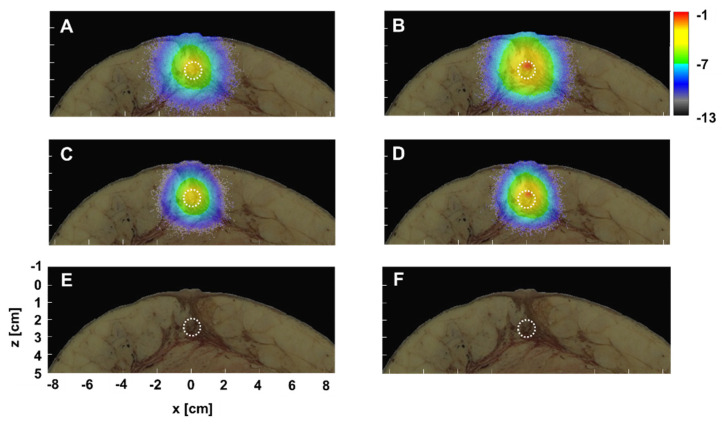
Effect of the excitation gradient in the SWIR (**A**,**B**), the NIR (**C**,**D**) and the VIS (**E**,**F**). Fluence maps without excitation gradient (**A**,**C**,**E**) and with gradient (**B**,**D**,**F**). Fluorescence-labeled cancer with a diameter of 1 cm in the duct were embedded *x* = 0 at a depth of 2 cm, denoted by circles of the white broken line. All maps share the values on the *x* and the *z*-axis of (**E**) and the scale bar, which shows the logarithmic scale of intensity (W/cm^2^) in (**B**).

In this study, the Monte Carlo model was developed with or without the excitation gradient (Figure 3). The effect of the excitation gradient on VIS, NIR, and SWIR was examined. Fluence maps without and with excitation gradient are shown in Figure 4 when fluorescence-labeled cancer of 1 cm diameter in the duct was embedded *x* = 0 at a depth of 2 cm. Because high *μ_s_* and *μ_a_* were obstructive against reaching fluorescence-labeled cancer for VIS excitation, there was little fluorescence, with and without excitation gradient. In contrast, since the excitation of NIR and SWIR reached the fluorescence-labeled cancer, there was fluorescence with and without excitation gradient. Furthermore, taking the excitation gradient into account, the upper area of cancer shows strong signals in both NIR and SWIR. Without excitation gradient, homogeneous signals in NIR and SWIR were distributed within the entire cancer. The fluence map of SWIR simulation with excitation gradient indicated near-surface signals stronger than those of the NIR. Therefore, the previous model was improved by the use of rational mechanisms for fluorescence, further confirmed in three regions, the VIS, the NIR, and the SWIR, in this study.

### 3.2. Setting Optical Parameters Faithful to Duct Morphology

In the previous model, spherically fluorescence-labeled cancer was embedded in the fat layer which had homogeneous optical parameters. However, the model was too simple as the actual breast tissue structure was complex. Breast cancer is frequently developed in the duct and progresses invasively. Thus, it is quite important to set optical parameters for ducts with complex morphology (Figure 1) when photon migration in the breast tissue is examined. Furthermore, the excitation and emission would be influenced by the various locations of fluorescence-labeled cancer within the duct, outside of the duct, or between the duct and the fat, due to the different optical properties that can be characteristic of each location (see Table 1).

In this study, optical parameters specific to various regions such as skin, duct, and fat were provided for the analytical model based on the breast structure data of the VHP. When the fluorescence-labeled cancer of 1 cm diameter was embedded in the duct at a depth of 2 cm, the fluorescent fluence signals from the spherical cancer had a small and vertically long shape in both the NIR and the SWIR, probably due to a higher *μ_s_* and a lower *μ_a_* of the duct shown in Figure 4A–D. The distortion from the perfect circle in the SWIR fluorescence signals was smaller than that in the NIR due to the low *μ_s_*. Moreover, the signal shape on the fluence map can be attributed to the distribution of the optical parameters.

**Figure 5:**
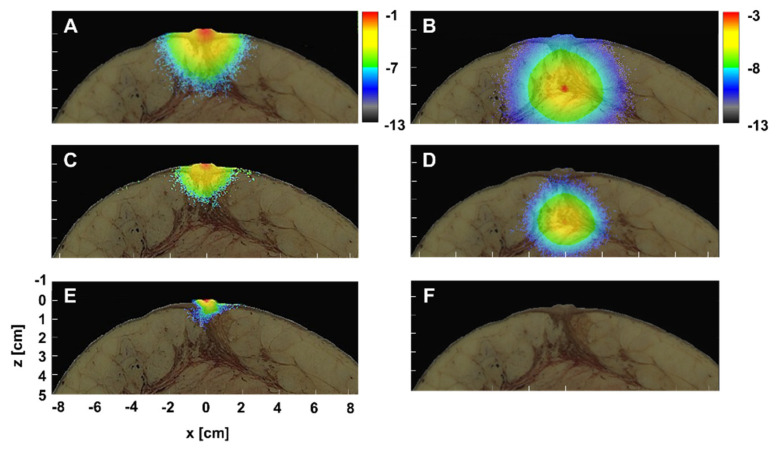
Fluence maps of photons associated with excitation (**A**,**C**,**E**) and emission (**B**,**D**,**F**) in the SWIR (**A**,**B**), the NIR (**C**,**D**), and the VIS (**E**,**F**). Fluorescence-labeled cancer with a diameter of 1 mm in the duct was embedded *x* = 0 at a depth of 3 cm. All maps share values on the *x* and *z*-axis of (**E**). (**A**,**C**,**E**) share the scale bar which shows the logarithmic scale in (**A**) and (**B**,**D**,**F**) share the scale bar in (**B**).

To confirm this possibility, the fluence map of excitation and emission were examined in detail when the spherical cancer of 1 mm diameter was embedded in the duct at a depth of 3 cm. Although there was no emission fluence of the VIS in Figure 4, we confirmed the effect on excitation of the faithful distribution of the optical parameters due to a higher *μ_s_* and *μ_a_* of the VIS than the NIR and the SWIR. As shown in Figure 5E, the VIS excitation fluence signals along the duct appeared due to the migration of photons, impeded by fat with a higher *μ_a_*. However, no photons reached the fluorescence-labeled cancer (1 mm) embedded in the deep region of the duct at a depth of 3 cm. Then, no fluorescence probes within cancer were excited by the incident photon in Figure 5F.

The excitation fluence signals of the NIR are distributed more widely than those of the VIS in Figure 5C. The signal shape of the excitation fluence of NIR was not isotropic and the signals were distributed a little downward. Although the incident photons excited the fluorescent probes within the cancer, the breast surface was not reached by any emission photon in Figure 5D. The distribution of the emission fluence signals was not isotropic either. In contrast to the excitation, the distribution was a little upward.

The excitation fluence signals of the SWIR were distributed widely than those of the NIR in Figure 5A. The distribution was downward and similar to that of the NIR. Sufficient incident photons from the SWIR reached the fluorescent probes within the cancer and emission photons reached the breast surface in Figure 5B. Therefore, the previous model was improved by a fluence signal distribution dependent on the contour of the breast tissue and the internal structure, and we confirmed this in three regions, VIS, NIR, and SWIR.

### 3.3. SWIR for the Detection of Small Breast Cancer in Deep Tissue

**Figure 6:**
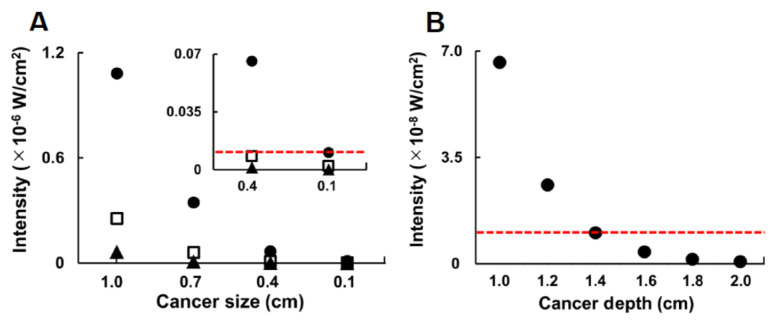
Effect on the detected fluorescence intensity of cancer size (**A**, 0.1–1.0 cm) and cancer depth (**B**, 1–2 cm). Insert is enlarged on the axis of the intensity. Closed circles: SWIR, Open squares: NIR, Closed triangles: VIS. The red broken line denotes the detectable intensity of 10 nW/cm^2^.

By the use of the improved Monte Carlo model improved above, photon behaviors in association with excitation and emission were examined in detail. Fluorescence-labeled cancer with a diameter of 1, 4, 7, and 10 mm was embedded at a depth of 1 to 2 cm. The fluorescence intensity detected on the tissue surface when the fluorescence-labeled cancer with a 1–10 mm diameter was embedded at a depth of 1 cm is shown in Figure 6A. In this study, a fluorescence intensity of more than 10 nW/cm^2^ was detectable and the sensitivity of fluorescence image sensors reported previously was taken as the basis of the assessment [28,29]. Fluorescence from cancerous cells with a diameter of 10 mm was detectable in all regions of the VIS, the NIR, and the SWIR. The SWIR signal was the strongest; the VIS was 5.9% of the SWIR, and the NIR it was 23.5% of the SWIR. In the case of the 7 mm diameter, there was almost no VIS signal and the NIR signal was 17.2% that of the SWIR. As shown in the insert, the signal of the NIR in the 4 mm diameter was under the detection limit and was 12.6% of the SWIR. In the case of the 1 mm diameter, only the SWIR signal was detectable.

Finally, as shown in Figure 6B, the detectable depth of the fluorescence from cancerous cells with a diameter of 4 mm was examined in detail. Due to the fact that no fluorescence from the VIS or the NIR from the depth of 1 cm was detectable, fluorescence-labeled cancer with SWIR alone was embedded deeper than 1 cm. At a depth of 1.2 cm, the fluorescence became weaker by 39.4% of that for 1 cm. The deepest position for cancer of a 4 mm diameter to be detectable was 1.4 cm. When the cancer was embedded deeper, the fluorescence was under the detection limit. Therefore, when the fluorescence-labeled cancer became smaller or was embedded in a deeper area, the fluorescence signal in all regions, the VIS, the NIR, and the SWIR, became weaker. However, fluorescence in the SWIR alone from cancerous cells with a diameter of 4 mm was detectable at a depth of 1.4 cm. The results show that SWIR fluorescence molecular imaging can be expected to detect breast cancer (4 mm), which is smaller than the cancer size (6 mm) detectable by MRI and sonography so far reported. Compared to the depth at which the diagnostic accuracy of sonography can be maintained (4 cm) [30], SWIR fluorescence molecular imaging may be more limited in detecting cancer, due to the depth. However, SWIR fluorescence molecular imaging has the potential advantage of distinguishing between benign and malignant tumors by the use of markers for the cancer. Furthermore, compared to MRI-sonography, the SWIR imaging system is expected to be the more practical diagnostic instrument with small size at a low running cost.

In addition to our SWIR molecular imaging for detecting early breast cancer, recently there have been remarkable advances in diagnostic methods. One of these was the early detection of circulating tumor cells in blood, an eagerly awaited noninvasive diagnosis or prognosis method [31]. In vitro assays confirmed the real time detection of cancer cells at 49 cells/mL [32]. Using a DNA biosensor based on gold nanoparticles-modified graphene oxide, breast cancer markers in the early stage were detected at a sub-nanomolar level [33]. Furthermore, when the ensemble discrete wavelet transformation as a new image processing technology was introduced to conventional mammography, the benign/malignant ROIs (regions of interest) were predicted at a precision rate of more than 97% by microcalcification cluster classification [34]. When the SWIR molecular imaging proposed in this study is applied clinically, it can be regarded as a noninvasive diagnostic method for primary breast cancer in women, without radiation exposure.

## 4. Conclusions and Perspectives

In the Monte Carlo model where optical parameters were set faithfully to the internal structure of the breast tissue, photon migration in association with excitation and emission was examined in detail based on the rational mechanisms for fluorescence. SWIR fluorescence molecular imaging proved the promising for the detection of small breast cancer (4 mm) at a depth of 1.4 cm, which was difficult for MRI and sonography to detect. Our results show that it is possible to predict the presence of early-stage breast cancer with high spatial resolution using SWIR and a phantom model in which the optical parameters are accurately set in the breast structure. The SWIR molecular imaging proposed in this study is suitable as a noninvasive diagnostic method for primary breast cancer in women, without radiation exposure.

## Figures and Tables

**Table 1:** Optical parameters specific to tissues in the visible (VIS), the near-infrared (NIR), and the shortwave-infrared (SWIR).

	Wavelength (nm)	Tissue	*µ_a_* (cm^−1^)	*µ_s_* (cm^−1^)	*n*
VIS	Ex 488	Skin	6.0	625	1.37
		Fat	6.0	310	1.45
		Duct	0.2	317	1.42
		Cancer	1.0	300	1.45
	Em 520	Skin	5.8	450	1.37
		Fat	4.0	300	1.45
		Duct	0.2	268	1.42
		Cancer	1.0	230	1.45
NIR	Ex 780	Skin	2.0	241	1.37
		Fat	1.4	136	1.45
		Duct	0.2	169	1.42
		Cancer	1.0	150	1.45
	Em 820	Skin	1.2	228	1.37
		Fat	1.2	132	1.45
		Duct	0.2	198	1.42
		Cancer	0.7	140	1.45
SWIR	Ex 970	Skin	1.0	210	1.37
		Fat	0.9	76.6	1.45
		Duct	0.3	122	1.42
		Cancer	0.9	75.5	1.45
	Em 1100	Skin	0.7	176	1.37
		Fat	0.6	72.3	1.45
		Duct	0.3	122	1.42
		Cancer	1.0	80.0	1.45
